# Discovery of a Ferroptosis-Related lncRNA–miRNA–mRNA Gene Signature in Endometrial Cancer Through a Comprehensive Co-Expression Network Analysis

**DOI:** 10.3390/curroncol33010037

**Published:** 2026-01-09

**Authors:** Hikaru Murakami, Junlong Wang, Herbert Yu

**Affiliations:** Cancer Epidemiology Program, University of Hawaii Cancer Center, 701 Ilalo St., Honolulu, HI 96813, USA; juwang@cc.hawaii.edu (J.W.); hyu@cc.hawaii.edu (H.Y.)

**Keywords:** endometrial cancer, ferroptosis, prognostic model

## Abstract

**Background**: As a newly recognized type of cell death implicated in cancer, ferroptosis plays multiple roles in tumor biology. Here, we sought to construct a prognostic framework for EC on the basis of ferroptosis-related long non-coding RNAs (FerlncRNAs), microRNAs (FermiRNAs), and mRNAs (FRGs) for endometrial cancer (EC). **Methods**: Transcriptomic profiles of tumors and matched clinical data for 544 EC patients were retrieved from TCGA-UCEC. A prognostic framework was generated through Cox regression, integrating ferroptosis-linked lncRNAs, miRNAs, and mRNAs. EC cases were stratified into groups with high or low predicted risk based on ferroptosis-related gene expression. The model’s prognostic utility was examined through Kaplan–Meier (K–M) analysis and receiver operating characteristic curves. **Results**: A prognostic model based on 16 RNAs, including 10 FerlncRNAs, 2 FermiRNAs, and 4 FRGs, was developed. Analysis using K–M plots showed that high-risk patients experienced shorter overall survival than their low-risk counterparts (*p* < 0.001). The model’s area under curve (AUC) values were 0.731, 0.749, and 0.768 at 1-, 3-, and 5-year time points, surpassing those of standard clinical parameters. Furthermore, in an external validation cohort, these signature RNAs were associated with EC prognosis. **Conclusions**: The novel ferroptosis-related lncRNA–miRNA–mRNA prognostic model provides a basis to assess clinical prognosis in EC patients.

## Simple Summary

Ferroptosis, a newly discovered form of cell death related to cancer, is regulated by mRNAs as well as non-coding RNA molecules, including long non-coding RNAs (lncRNAs) and microRNAs (miRNAs). However, examination of ferroptosis within endometrial cancer (EC) remains limited. In the current investigation, we used publicly available data for 521 patients with EC and identified 16 signal ferroptosis-related RNA molecules containing 10 lncRNAs, 2 miRNAs, and 4 mRNAs, associated with prognosis. A predictive system for patient prognosis on the basis of these ferroptosis-associated RNAs was developed. Notably, this prognostic framework exhibited superior predictive accuracy for EC prognosis, with a higher AUC value than that of conventional clinical factors, containing diagnostic age, tumor differentiation, and stage classification. This study is the first to propose a comprehensive ferroptosis-associated lncRNA–miRNA–mRNA regulatory circuitry in EC. The present findings offer a robust basis for future analyses of ferroptosis in EC.

## 1. Introduction

Among women in developed nations, endometrial cancer (EC) is the most frequent gynecological cancer and globally ranks sixth in incidence [1]. Due to falling birthrate and rising prevalence of obesity, its incidence has risen in recent years, including among younger individuals [2]. EC treatment involves multiple approaches, including surgery, chemotherapy, radiation therapy, and targeted therapies, depending on the histopathologic features and clinical presentation [3]. Most patients are diagnosed with well-differentiated endometrioid adenocarcinoma, which tends to be detected at an early stage and has a favorable prognosis [4]. Despite being classified as low-grade, early-stage, and well-differentiated, certain endometrioid tumor cases recur and are associated with adverse outcomes [5]. Clinical outcomes are considerably poorer in patients with advanced or recurrent tumors than in those with early-stage EC who remain recurrence-free [6]. Therefore, reliable prognostic biomarkers are urgently needed for the selection of optimal treatment strategies and identification of new therapeutic targets.

As a recently identified regulated cell death pathway, ferroptosis is distinct in both mechanism and biochemistry from apoptosis, necrosis, and autophagy. It is defined by iron-dependent lipid peroxidation driven by dysregulated redox homeostasis and the accumulation of reactive oxygen species (ROS) at cellular membranes [7,8]. It acts as an intrinsic tumor-suppressive mechanism by limiting cancer cell proliferation and invasion, and it modulates the tumor microenvironment and resistance to therapy, suggesting that the dysregulation of ferroptosis contributes to carcinogenesis and tumor progression [9]. Approximately 500 ferroptosis genes, represented by glutathione peroxidase 4 (*GPX4*), have been discovered [10]. Despite increasing evidence for the roles of ferroptosis in carcinogenesis as well as tumor progression and suppression [11,12], research on ferroptosis in EC remains limited.

Non-coding RNAs (ncRNAs) are RNA transcripts that do not encode proteins. Although approximately 95–98% of the human genome does not encode proteins, only a subset of non-coding regions is transcribed into various classes of ncRNAs, playing central roles in the regulation of genes [13]. Within ncRNAs, long non-coding RNAs (lncRNAs), which are transcripts exceeding 200 nucleotides in length [14], and microRNAs (miRNAs), which are 21–25 nucleotides [15], play important roles in ferroptosis [16,17]. In particular, lncRNAs and miRNAs coordinately modulate ferroptosis-related mRNAs via competing endogenous RNA networks [18]. Regulatory networks of ferroptosis-related lncRNAs, miRNAs, and mRNAs have been reported in some cancer types [19,20]. However, the regulatory interactions among ferroptosis-related lncRNAs, miRNAs, and mRNAs in EC have yet to be investigated. Therefore, analyses of lncRNA–miRNA–mRNA signatures are needed to capture the ferroptosis-driven regulatory circuitry and its prognostic relevance in EC.

In the present work, we identified ferroptosis-associated mRNAs from a ferroptosis database and analyzed their correlations with lncRNAs and miRNAs and differential expression in EC using The Cancer Genome Atlas (TCGA) data. Next, a ferroptosis-associated lncRNA–miRNA–mRNA correlation map was generated. We developed a ferroptosis-related lncRNA–miRNA–mRNA model for forecasting overall survival (OS) in individuals with EC. We developed a nomogram incorporating the ferroptosis-associated lncRNA–miRNA–mRNA signature to achieve a more comprehensive understanding of the molecular and signaling pathways underlying ferroptosis in EC. Our study provides a basis for predicting OS and is expected to guide treatment and further research on the regulatory network in EC.

## 2. Materials and Methods

### 2.1. Compilation of Data

Individuals with a pathological diagnosis of uterine corpus endometrial carcinoma (UCEC) up to 3 August 2024, were enrolled; their RNA-seq results and clinical data were extracted from TCGA-UCEC “https://xenabrowser.net/datapages/ (accessed on 10 August 2024)”. After removing cases with incomplete clinical information, data for 544 patients with EC were retained. Clinical information for patients with EC included mortality status, diagnostic age, clinical stage, pathological grade, and total survival period. Additionally, RNA sequencing information from 23 normal endometrial tissue samples was collected from the TCGA-UCEC database. Transcriptomic profiles and corresponding clinical annotations for 213 EC cases were obtained from the Clinical Proteomic Tumor Analysis Consortium (CPTAC) through the University of California Santa Cruz (UCSC) Xena platform “https://xenabrowser.net/datapages/ (accessed on 29 November 2024)” for validation purposes.

### 2.2. Identification of FerlncRNAs and FermiRNAs

We obtained 489 ferroptosis-related genes (FRGs) from the FerrDb “https://www.zhounan.org/ferrdb/v3/pages/index.html (accessed on 20 August 2024)”, a comprehensive platform for ferroptosis-associated gene annotation. For the screening of ferroptosis-related lncRNAs (FerlncRNAs) and ferroptosis-related miRNAs (FermiRNAs), we estimated Pearson correlation coefficients to determine relationships between FRGs and lncRNAs as well as between FRGs and miRNAs in the TCGA dataset. LncRNAs and miRNAs with |*R*| > 0.35 and *p* < 0.001 were retained for subsequent analyses. These thresholds were adopted based on a previous study [21].

### 2.3. Identification of DEFRGs, DEFerlncRNAs, and DEFermiRNAs

We employed the “limma” package in R software (version 4.3.3; R Foundation for Statistical Computing, Vienna, Austria) to detect differentially expressed FRGs (DEFRGs), differentially expressed FerlncRNAs (DEFerlncRNAs), and differentially expressed FermiRNAs (DEFermiRNAs) between tumor samples from EC and noncancerous endometrial tissues (adjusted *p*-value < 0.05, |log fold change [log FC]| > 1).

### 2.4. Identification of Signal Ferroptosis-Related RNAs

Univariate Cox regression analyses of DEFRGs, DEFerlncRNAs, and DEFermiRNAs were performed to screen for genes strongly related to survival outcomes in EC cases (*p* < 0.1). Subsequently, regression coefficients for the prognostic model were calculated using multivariate Cox regression analysis. We selected 16 signal ferroptosis-related RNAs, including 10 lncRNAs, two miRNAs, and four mRNAs, to predict OS in EC. To reveal functional protein networks among signature FRGs, we constructed a protein–protein interaction (PPI) network of the four FRGs utilizing the STRING platform “https://cn.string-db.org/ (accessed on 11 November 2024)” [22]. In addition, we drew a map of the correlation network for signature FRGs, in which the correlations among signature FRGs were calculated using Pearson correlation analyses. Moreover, we created a comprehensive ferroptosis-related lncRNA–miRNA–mRNA correlation map.

### 2.5. Establishment of a Prognostic Model Based on the Ferroptosis-Related lncRNA-miRNA-mRNA Signature

A prognostic risk score was generated from the expression of 16 ferroptosis-associated RNAs. Patient-specific risk scores were derived according to methods reported in previous studies, as shown in the following formula [23,24]:

$$\mathrm{Risk}\;\mathrm{score}\;=\;\sum_{i=1}^{n}\beta i\;\times \;Exp\;(i)$$

Using the median risk score as a cutoff, EC patients were stratified into high- and low-risk categories. Expression patterns of the ferroptosis-associated RNAs in the two groups were visualized with a heatmap generated via the “pheatmap” package in R. We also generated a risk profile curve along with scatter plots of survival outcomes. Kaplan–Meier (K–M) survival curves were generated and compared using the log-rank test to assess differences in OS between the two groups, with analyses performed using the “survminer” package in R. The prognostic model’s predictive accuracy was assessed using time-dependent receiver operating characteristic (ROC) curves generated with the “survivalROC” package in R. Additionally, a principal component analysis (PCA) and t-distributed stochastic neighbor embedding (t-SNE) analyses were conducted using the “stats” and “Rtsne” packages in R, respectively, for dimensionality reduction in the signal RNAs.

### 2.6. Verification of the Signal Ferroptosis-Related RNAs Using the CPTAC Cohort

To externally validate the 16 signature ferroptosis-related RNAs, an independent validation cohort of 213 EC cases from CPTAC was analyzed. Univariate Cox analyses were applied to evaluate the relationship between the signature RNAs and OS. The cohort of 213 cases was separated into two or three subgroups according to diagnostic age, tumor differentiation, or clinical stage, and the 16 signature RNAs’ expression levels were evaluated among these subgroups. Statistical differences between two groups were determined using the Mann–Whitney U test, and differences among three groups were assessed with the Kruskal–Wallis test. The association between expression levels of each of the 16 signature RNAs and OS was assessed using K–M curves with log-rank tests.

### 2.7. Development of the Prognostic Scoring Nomogram

Univariate and multivariate Cox regression analyses were performed to evaluate whether our prognostic model served as an independent predictor of mortality in EC after adjusting for conventional clinical variables, including diagnostic age, tumor differentiation, and clinical stage. Forest plots were generated utilizing the “limma” and “ggpubr” packages in R. Using the “rms” package in R, a nomogram was built. Calibration plots were generated to examine the agreement between model predictions and actual observations. The performance of the model was assessed in terms of sensitivity and specificity using ROC curve analysis. The model’s potential clinical value was assessed through decision curve analysis (DCA) across a spectrum of threshold probabilities.

### 2.8. GSEA

Using gene set enrichment analysis (GSEA) software (version 4.3.3; Broad Institute, Cambridge, MA, USA), we performed GSEA to compare pathway activity between the two risk groups. For this analysis, we utilized the “c2.cp.kegg.v7.4.symbols.gmt” gene set in the Molecular Signatures Database “http://www.gsea-msigdb.org/ (accessed on 5 April 2025)”.

### 2.9. Statistical Evaluation

All data analyses were conducted with R software, version 4.3.3. Pearson’s correlation analyses were used to evaluate correlations. Comparisons of quantitative variables between two groups were performed using the Mann–Whitney U test. The Kruskal–Wallis test was used for comparisons among three groups. K–M survival curves were generated to visualize differences in survival, and curves were compared using the log-rank test. Two-tailed *p*-values were applied for all statistical analyses, and significance was defined at a threshold of *p* < 0.05 unless noted otherwise.

## 3. Results

### 3.1. Screening for DEFRGs, DEFerlncRNAs, and DEFermiRNAs

Transcriptomic analysis of 544 EC cases revealed 13,860 lncRNAs and 1447 miRNAs. Using 489 FRGs from FerrDb, FerlncRNAs and FermiRNAs were screened. A total of 8842 lncRNAs and 596 miRNAs exhibited significant associations with the expression of FRGs. Of these, 87 FerlncRNAs and 13 FermiRNAs exhibited significant differential expression in comparisons of tumor samples versus noncancerous endometrial tissues. In addition, 19 DEFRGs were detected when comparing EC tumors with noncancerous uterine endometrium.

### 3.2. Identification of Signal Ferroptosis-Related RNAs and Construction of PPI and Correlation Networks

**Figure 1:**
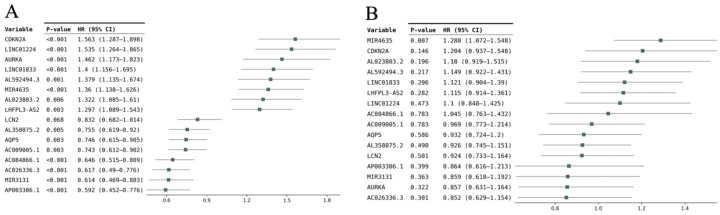
Discovery of signal ferroptosis-related RNAs. (**A**) Forest plot showing the outcomes of univariate Cox analyses; (**B**) Forest plot showing the outcomes of a multivariate Cox analysis. HR, hazard ratio; CI, confidence interval.

**Figure 2:**
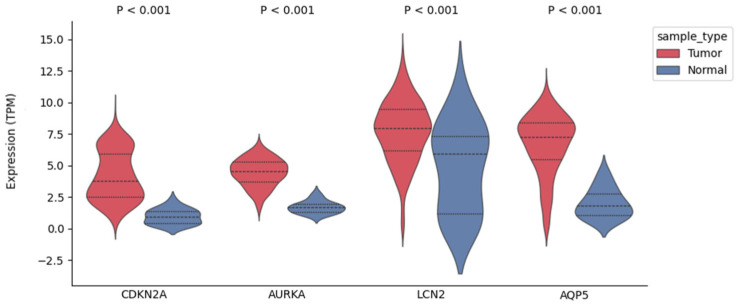
Comparison of the expression levels of the four signal ferroptosis-related mRNAs between EC tissues and normal endometrium. EC, endometrial cancer; TPM, transcripts per million.

**Figure 3:**
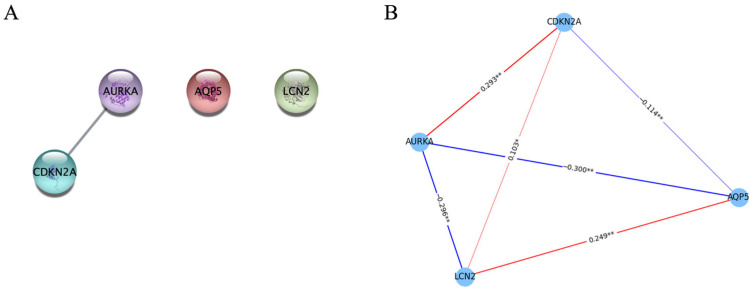
Visualization of the relationships among four significant ferroptosis-related mRNAs. (**A**) PPI network of the four ferroptosis-associated mRNAs. Nodes represent ferroptosis-associated mRNAs, and edges indicate predicted protein–protein interactions; (**B**) Correlation network based on Pearson correlation analyses. Nodes represent the four signal ferroptosis-related genes. The red and blue lines correspond to positive and negative correlations, respectively. Line thickness reflects the absolute value of the Pearson correlation coefficient (the thicker the line, the larger the value). PPI, protein–protein interactions. *: *p* < 0.05, **: *p* < 0.01.

Using univariate Cox analyses, we evaluated the 87 DEFerlncRNAs, 13 DEFermiRNAs, and 19 DEFRGs. Ten lncRNAs, two miRNAs, and four mRNAs were identified as significant ferroptosis-related RNAs (*p* < 0.1, Figure 1A). These RNAs were ultimately analyzed using multivariate Cox regression to derive the coefficients incorporated into the prognostic model (Figure 1B). The four FRGs were as follows: cyclin-dependent kinase inhibitor 2A (*CDKN2A*), aurora kinase A (*AURKA*), lipocalin 2 (*LCN2*), and aquaporin 5 (*AQP5*). As shown in Figure 2, the expression of all four FRGs was markedly elevated in EC tissues relative to normal endometrium. Figure 3A,B illustrate the PPI and correlation networks of the four FRGs, respectively. In the PPI network, a correlation was observed between AURKA and CDKN2A (confidence score > 0.4). As shown in the correlation network, we observed positive correlations between AURKA and CDKN2A, AQP5 and LCN2, and CDKN2A and LCN2, as well as negative correlations between AQP5 and AURKA, AURKA and LCN2, and AQP5 and CDKN2A.

### 3.3. Establishment of a Co-Expression Network of Ferroptosis-Associated lncRNAs, miRNAs, and mRNAs

**Figure 4:**
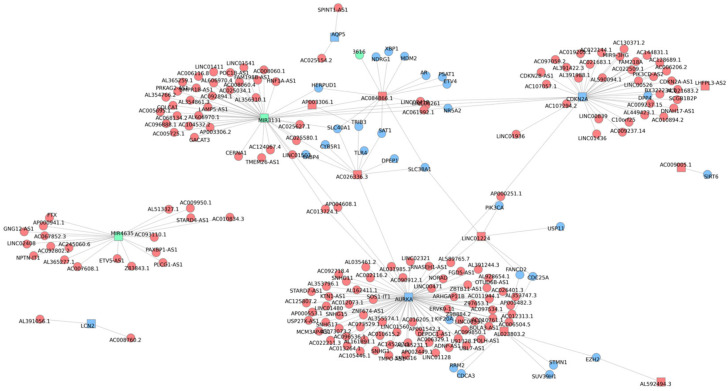
Comprehensive co-expression network of the ferroptosis-associated lncRNAs, miRNAs, and mRNAs. Nodes represent RNAs, with different colors indicating RNA types (red for lncRNAs, green for miRNAs, and blue for mRNAs). Square nodes denote the 16 signal ferroptosis-related RNAs included in the prognostic model. Edges represent significant correlations (|*R*| > 0.4, *p* < 0.05). LncRNA, long non-coding RNA; miRNA, microRNA.

**Figure 5:**
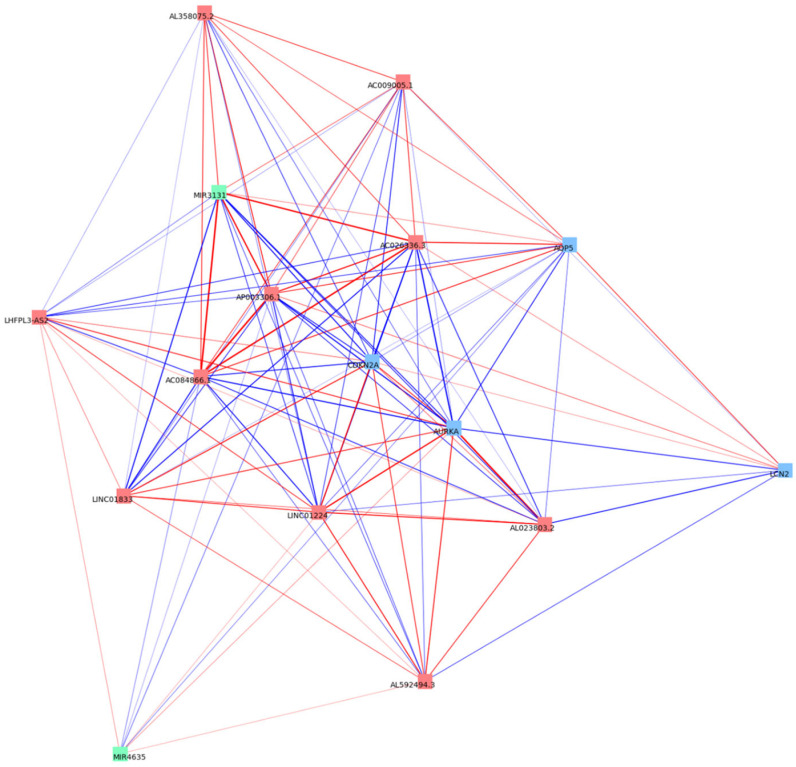
Correlation network of 16 signal ferroptosis-related RNAs. Nodes represent RNAs, with different colors indicating RNA types (red for lncRNAs, green for miRNAs, and blue for mRNAs). Red and blue edges denote positive and negative correlations, respectively, and edge thickness corresponds to the absolute correlation strength. LncRNA, long non-coding RNA; miRNA, microRNA.

A comprehensive correlation network of the ferroptosis-associated lncRNA–miRNA–mRNA genes is displayed in Figure 4 (|*R*| > 0.4, *p* < 0.05). Notably, miR-3131 and AC026336.3 correlated with solute carrier family 40 member 1 (*SLC40A1*), which is crucial for controlling intracellular Fe^2+^ concentrations; LINC01224 was correlated with fanconi anemia complementation group D2 (*FANCD2*), a major regulator of ferroptosis through the modulation of GPX4; and *CDKN2A* was correlated with dipeptidyl peptidase 4 (*DPP4*), which contributes to ROS accumulation. Furthermore, a correlation network of the signature ferroptosis-related lncRNA–miRNA–mRNA genes is displayed in Figure 5. Strong correlations were observed between AC026336.3 and miR-3131, AC026336.3 and CDKN2A, and miR-3131 and CDKN2A.

### 3.4. Analysis of Patients with EC Stratified by the Ferroptosis-Related RNA Risk Score

**Figure 6:**
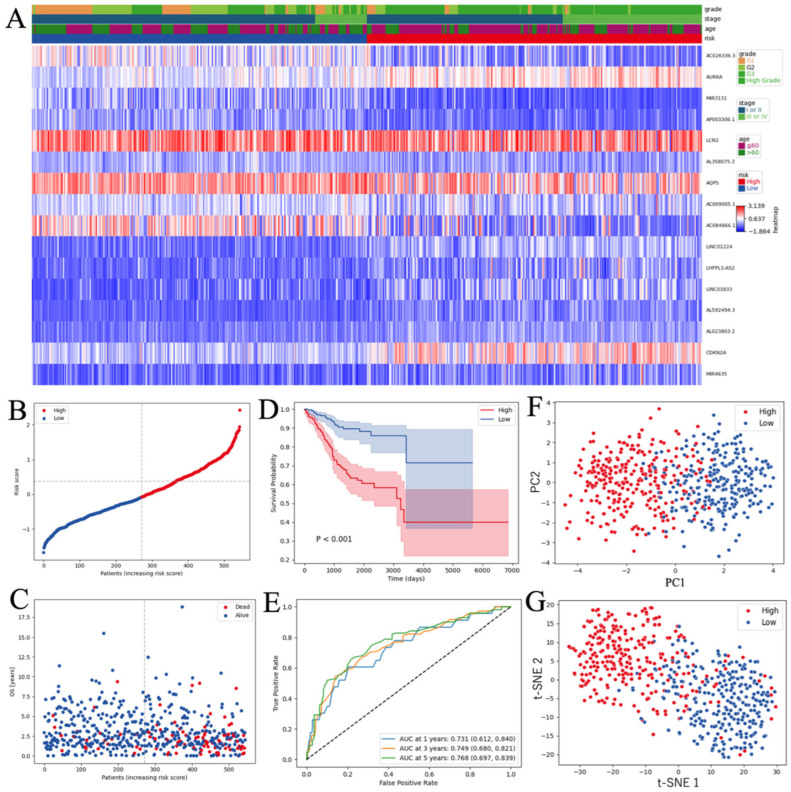
Evaluation of the prognostic performance of the ferroptosis-associated RNAs model in TCGA (**A**) Expression heatmap of the 16 signature ferroptosis-associated RNAs; (**B**) Risk score distribution; (**C**) Survival status scatter plots; (**D**) K–M survival curves for patients in the high- and low-risk groups; (**E**) Time-dependent ROC curve analysis; (**F**) PCA based on the expression profiles of the 16 signal ferroptosis-related RNAs. Each point represents one patient with EC. The percentages on the axes indicate the proportion of variance explained; (**G**) t-SNE analysis showing the separation of patients based on the same expression profiles. TCGA, The Cancer Genome Atlas; K–M, Kaplan–Meier; ROC, receiver operating characteristic; PCA, principal components analysis; EC, endometrial cancer; t-SNE, t-distributed stochastic neighbor embedding.

EC cases were stratified into high- and low-risk categories according to their risk scores, with the median value used to separate the groups. The heatmap depicted significant upregulation of eight ferroptosis-related RNAs (CDKN2A, LINC01224, AURKA, LINC01833, AL592494.3, MIR4635, AL023803.2, and LHFPL3-AS2) and downregulation of eight ferroptosis-related RNAs (LCN2, AL358075.2, AQP5, AC009005.1, AC084866.1, AC026336.3, miR-3131, and AP003306.1) in patients classified as high-risk versus those classified as low-risk (Figure 6A). Patients in the high-risk category exhibited increased mortality relative to low-risk patients (Figure 6B,C). Furthermore, a K–M survival analysis revealed a significantly poorer OS among high-risk patients compared with low-risk patients (*p* < 0.001, Figure 6D). A time-dependent ROC analysis demonstrated that, for 1-, 3-, and 5-year survival, our model achieved area under the curve (AUC) values of 0.731, 0.749, and 0.768, respectively (Figure 6E). PCA and t-SNE results supported the validity of the grouping strategy (Figure 6F,G).

### 3.5. External Validation of the Signal Ferroptosis-Related RNAs in CPTAC

**Figure 7:**
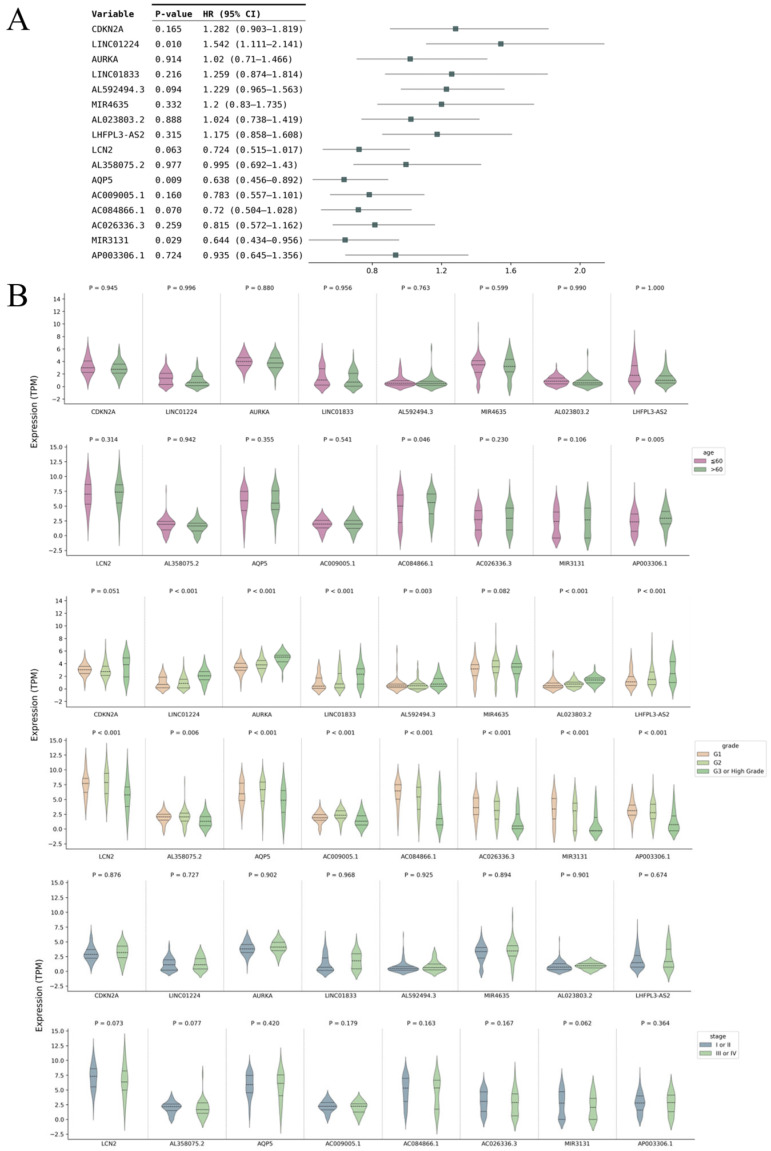
Validation of the 16 signal ferroptosis-related RNAs in CPTAC. (**A**) Forest plot illustrating the outcomes of univariate Cox analysis of the 16 signature RNAs; (**B**) Expression comparison of the 16 signature RNAs in patient subgroups based on age, grade, and stage. CPTAC, Clinical Proteomic Tumor Analysis Consortium; HR, hazard ratio; CI, confidence interval; TPM, transcripts per million.

**Figure 8:**
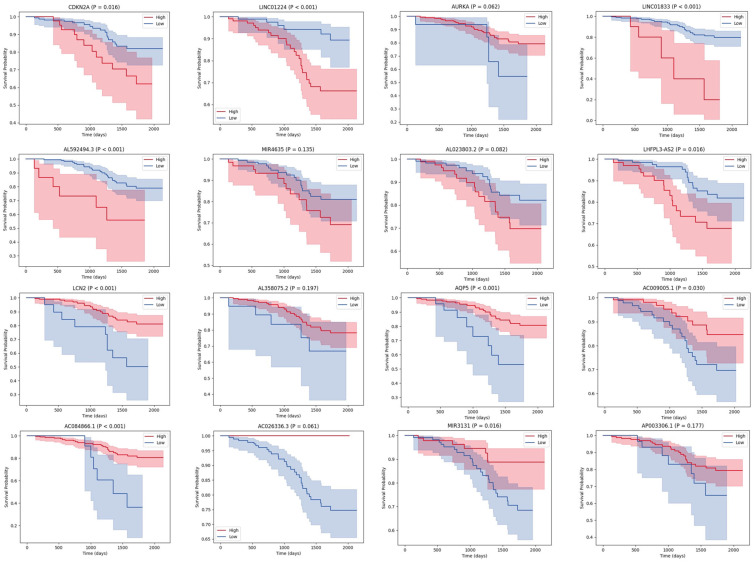
K–M survival curves of the 16 signature RNAs and overall survival in the CPTAC cohort. K–M, Kaplan–Meier; CPTAC, Clinical Proteomic Tumor Analysis Consortium.

The 16 signature ferroptosis-related RNAs were validated using data for patients with EC in CPTAC. Univariate Cox regression analyses revealed that the hazard ratio directions for all 16 signature RNAs were concordant between the TCGA and CPTAC datasets (Figure 7A). In addition, the associations between each signature RNA and clinical characteristics were examined. Almost all signature RNAs showed significant associations with tumor grade, and the directions of expression changes aligned with the TCGA results (Figure 7B). The survival curves corresponding to each signature RNA are presented in Figure 8. For all signal RNAs except AURKA, the differences in survival time between high and low expression were similar to those observed in TCGA. Thus, the findings from the CPTAC cohort were in agreement with those of the TCGA analyses, supporting the robustness of variable screening and coefficient determination for our model.

### 3.6. Independent Predictive Value of the Signature Ferroptosis-Associated RNA Model and Nomogram Establishment

**Figure 9:**
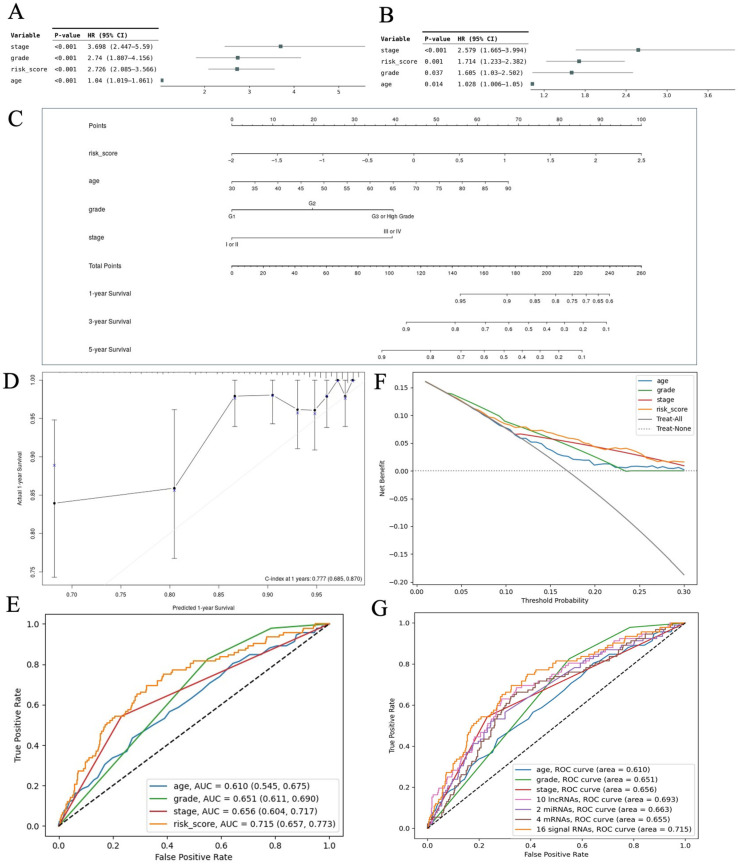
Association between the risk score and clinical features. (**A**) Forest plot showing the results of univariate Cox analyses; (**B**) Forest plot showing the results of a multivariate Cox analysis; (**C**) Nomogram incorporating the risk score and clinicopathological factors; (**D**) Calibration curve of the nomogram for survival prediction; (**E**) Comparison of AUC values between the risk model and clinicopathological models using multi-index ROC analysis; (**F**) DCA of the risk score and clinicopathological variables; (**G**) Comparison of AUC values between the risk score-based prognostic models and clinical index-based prognostic models using a multi-index ROC analysis. AUC, area under the ROC curve; ROC, receiver operating characteristic; DCA, decision curve analysis; HR, hazard ratio; CI, confidence interval.

To determine whether our model independently predicted prognosis in EC patients, univariate and multivariate Cox analyses were conducted incorporating the risk score and clinicopathological variables, including diagnostic age, histological grade, and clinical stage. Univariate analyses demonstrated that OS was significantly related to age, grade, stage, and risk score (Figure 9A). Moreover, the multivariate analysis showed that OS was independently associated with age, grade, stage, and risk score (Figure 9B). The prognostic nomogram was designed to predict the probability of OS at 1, 3, and 5 years. Figure 9C demonstrated that the risk score was a major determinant of OS. Good concordance between the predicted and actual probabilities was observed in the calibration curve (Figure 9D). The ROC analysis demonstrated that our model achieved an AUC of 0.715, surpassing the prognostic performance of any clinicopathological variable (Figure 9E). DCA further demonstrated that our model provided reliable predictive performance and meaningful clinical utility (Figure 9F). Furthermore, the ferroptosis-associated RNA signature had higher prognostic value than those of other risk models based only on the signature of 10 lncRNAs, two miRNAs, or four mRNAs (Figure 9G).

### 3.7. Comparison of Enriched Biological Pathways Between High-Risk and Low-Risk Groups

**Figure 10:**
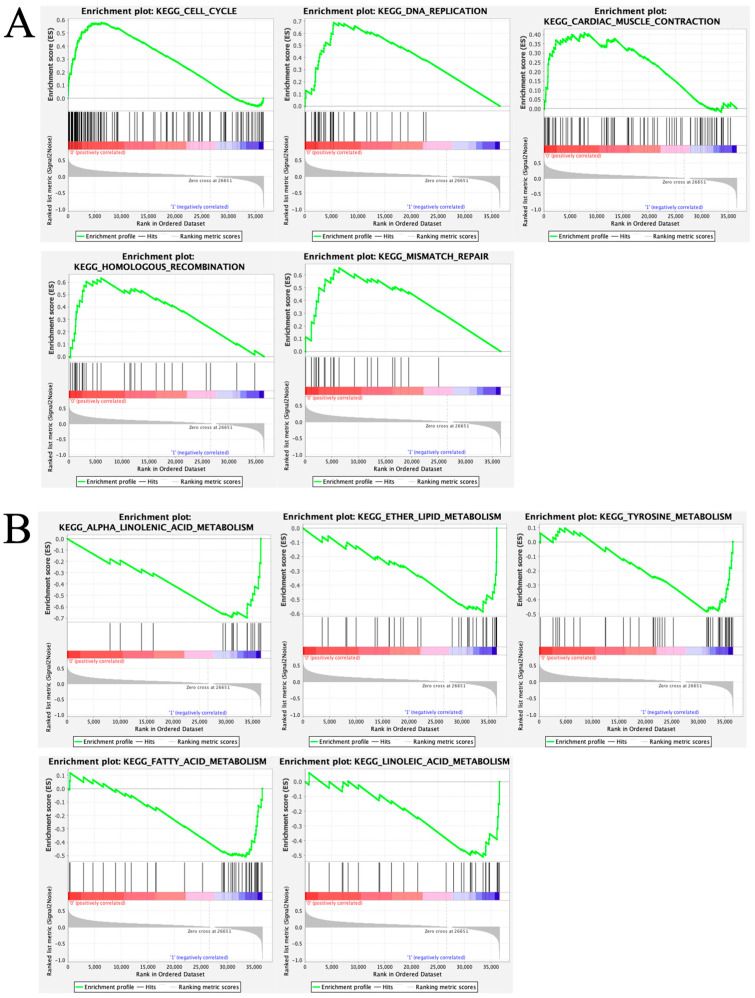
Comparison of high- and low-risk groups using GSEA. (**A**) GSEA-identified pathways with significant enrichment in the high-risk group; (**B**) GSEA-identified pathways with significant enrichment in the low-risk group. GSEA, Gene Set Enrichment Analysis.

To identify enriched pathways, GSEA was conducted using genes showing differential expression between high- and low-risk patients. The results showed significant enrichment for cell cycle, DNA replication, cardiac muscle contraction, homologous recombination, and mismatch repair in the high-risk group (Figure 10A). The low-risk group showed significant enrichment for alpha linolenic acid, ether lipid, tyrosine, fatty acid, and linoleic acid metabolism (Figure 10B).

## 4. Discussion

In this study, 16 significant ferroptosis-related RNAs, including 10 lncRNAs, two miRNAs, and four mRNAs, were identified through a transcriptomic analysis of the TCGA-UCEC cohort. The ferroptosis-associated RNA–based predictive model demonstrated a strong ability to predict prognosis in patients with EC. Subsequently, patients were stratified into high- and low-risk groups according to the prognostic model-derived risk scores, and differences in prognosis and enriched biological pathways between the two groups were evaluated. The findings indicated that high-risk EC patients had reduced OS relative to low-risk patients and showed enrichment of cancer-associated pathways, such as cell cycle, DNA replication, homologous recombination, and mismatch repair. Cell cycle- and DNA repair-associated pathways, including DNA replication, homologous recombination, and mismatch repair, contribute to ferroptosis by modulating proliferative stress, redox imbalance, and vulnerability to lipid peroxidation, thereby linking classical DNA damage responses with ferroptosis-associated oxidative stress pathways [25,26]. In clinical practice, our nomogram may assist clinicians in estimating individualized survival probabilities by integrating the ferroptosis-associated risk score with conventional factors. Compared with existing prognostic models based solely on clinicopathological variables, our model demonstrates improved predictive accuracy, suggesting potential value for risk stratification and treatment decision support in EC. Notably, the lncRNA–miRNA–mRNA integrated model showed better diagnostic value than that of any lncRNA-, miRNA-, or mRNA-based model. This is consistent with a previous report [27]; compared with single-level models based solely on lncRNAs, miRNAs, or mRNAs, the integrated lncRNA–miRNA–mRNA framework captures complementary multi-tiered regulatory connections across transcriptomic layers, minimizing information loss and enhancing prognostic accuracy. This multi-omics strategy enables a more robust representation of ferroptosis-related regulatory networks underlying EC progression. Moreover, we performed external validation of the 16 signature ferroptosis-related RNAs, supporting the relationships between these RNAs and prognosis. Thus, the model demonstrated high accuracy as an independent predictor of prognosis in EC.

In the current investigation, we established a co-expression map of ferroptosis-related lncRNA–miRNA–mRNA genes associated with prognosis in EC. The four FRGs were *CDKN2A*, *AURKA*, *LCN2*, and *AQP5*. *CDKN2A* has various roles in cancer. For example, earlier research has demonstrated that CDKN2A functions as a regulator of cell cycle checkpoints and autophagy, and its increased expression leads to poor prognosis in EC [28,29]. Yong et al. have shown that CDKN2A inhibits ferroptosis, influencing cisplatin resistance in cervical cancer [30]. Moreover, Liu et al. have established a predictive framework built on the basis of three ferroptosis-associated mRNAs, including *CDKN2A*, in colorectal cancer. Their study revealed that elevated CDKN2A expression is strongly linked to poor prognosis in colorectal cancer, suggesting that CDKN2A could serve as a novel prognostic and diagnostic biomarker as well as a potential therapeutic target [31]. These reports support the results of our study focused on EC. However, only a limited number of studies have investigated the function of CDKN2A in EC, and more investigations are required. AURKA belongs to the serine/threonine kinase family and is essential for cell division through its role in mitotic regulation. AURKA may promote carcinogenesis via cell proliferation, ferroptosis, and cancer stem cell self-renewal [32]. Jian et al. have indicated that the lncRNA SOCS2-AS1 inhibits EC advancement by controlling AURKA degradation [33]. Yuan et al. have shown that the AURKA inhibitor Alisertib might be effective in EC treatment, suggesting AURKA as a promising candidate for targeted therapy [34]. Guo et al. have reported that AURKA serves as a ferroptosis suppressor by upregulating GPX4, and its inhibition promotes ferroptosis, which enhances the effect of radiotherapy on breast cancer [35]. Thus, ferroptosis-related AURKA is commonly overexpressed across multiple cancer types, and elevated levels are linked to unfavorable clinical outcomes. In our study, we found a similar association between survival and AURKA expression in TCGA but not in CPTAC. This discrepancy could be attributable to the relatively small sample size and limited number of death events in the CPTAC cohort and to cohort-related differences such as patient composition, follow-up duration, and data generation platforms. In addition, AURKA-related biological effects may be context-dependent and influenced by tumor heterogeneity. Therefore, further studies with larger, well-annotated cohorts and extended follow-up are required to clarify the role of ferroptosis-associated AURKA in EC. LCN2 is a recently identified member of the lipocalin superfamily that possesses multiple functional roles [36]. The contribution of LCN2 to tumor biology varies across different types of cancer [37]. According to Huang et al., in pancreatic, ovarian, and hepatocellular carcinomas, LCN2 expression is markedly elevated during tumor initiation and subsequently declines as tumors progress, suggesting that LCN2 exerts a suppressive effect in the progression of tumors consistent with its expression pattern [37]. In the present study, LCN2 expression was elevated in EC tissues compared with normal endometrium and was inversely associated with OS, in line with earlier studies. Several reports have explored the contribution of LCN2 to ferroptosis in EC. As LCN2 regulates ferroptosis, serum levels have been tested for the diagnosis of EC [38]. LCN2 expression is also a potential marker for the evaluation of drug resistance in EC [39]. Moreover, Huang et al. have discovered five ferroptosis-related mRNAs with prognostic value in EC, including *CDKN2A* and *LCN2* [40]. Notably, despite the use of different datasets and statistical methods, *CDKN2A* and *LCN2* were identified as FGRs with prognostic value for EC in both our study and Huang et al. [40]. AQP5 belongs to the family of small hydrophobic proteins that function as androgen-regulated integral membrane water channels, playing roles in cellular water balance and growth signaling [41]. AQP5 is involved in regulating vascular permeability and interstitial fluid pressure within tumors [42]. Studies have shown that AQP5 influences tumor growth and may contribute to cancer metastasis and cell migration by increasing permeability and promoting the formation of cell projections [43,44]. Furthermore, AQP5 can induce ferroptosis by facilitating H_2_O_2_ uptake at the plasma membrane [45]. Increased intracellular H_2_O_2_ causes lipid peroxidase accumulation, mitochondrial destruction, and a decrease in GPX4, promoting ferroptosis. Thus, it is assumed that AQP5 is involved in the cancer process by regulating ferroptosis; however, the role of ferroptosis-related AQP5 has not been evaluated in EC. In this study, several key RNAs were correlated with each other. Of note, we detected strong correlations between AC026336.3 and miR-3131, AC026336.3 and CDKN2A, and miR-3131 and CDKN2A, suggesting the existence of an AC026336.3/miR-3131/CDKN2A pathway. Among the signal FRGs, CDKN2A showed the most significant association with prognosis in EC. Similarly, miR-3131 had the most significant association with EC prognosis among the signal FermiRNAs. Therefore, further investigations of this pathway may be promising. However, the proposed interactions are still hypothesis-generating. Although sequence-based evidence for direct miRNA–mRNA and miRNA–lncRNA interactions is critical for validating regulatory mechanisms, such analyses were not performed in the present investigation and should be examined in subsequent research to further elucidate the molecular basis of the proposed network. This work represents the first attempt to propose a ferroptosis-associated lncRNA–miRNA–mRNA co-expression network in EC. The limited number of ferroptosis-related mRNAs in the network reflected the application of stringent statistical and prognostic filtering criteria rather than a lack of biological relevance, highlighting the potential regulatory importance of ncRNAs in ferroptosis-related mechanisms.

This study had several limitations. Firstly, the use of TCGA data alone for building the risk prediction system could restrict the applicability of our results. Secondly, the availability of public transcriptomic datasets for non-coding RNAs is restricted. Although the CPTAC cohort provided independent validation, the modest number of death events and limited follow-up duration might have diminished the statistical power and sensitivity of survival analyses, potentially attenuating the strength of observed associations. Thirdly, all conclusions in this study were based solely on statistical analyses of publicly available data, and no experiments were performed. Therefore, prospective, high-quality, multicenter studies with large sample sizes, adequate follow-up, and laboratory experiments are needed to confirm our findings.

## 5. Conclusions

We established a new prognostic framework incorporating a ferroptosis-associated lncRNA–miRNA–mRNA gene signature through interrogating the transcriptomes of patients with EC. Using this model, we may improve the prediction of EC prognosis. This study introduces, for the first time, integrated co-expression networks of ferroptosis-associated lncRNAs, miRNAs, and mRNAs relevant to patient prognosis in EC. Since this study is based entirely on computational analyses, the findings cannot yet be applied in a clinical context. Nonetheless, this newly discovered network offers fresh perspectives on the molecular mechanisms of EC progression and potential therapeutic strategies.

## Data Availability

All primary data presented in this study are provided in the article, and any further questions should be directed to the corresponding author.

## Abbreviations

The following abbreviations are used in this manuscript:

EC	Endometrial cancer
ROS	Reactive oxygen species
GPX4	Glutathione peroxidase 4
ncRNA	Non-coding RNA
lncRNA	Long non-coding RNA
miRNA	MicroRNA
TCGA	The Cancer Genome Atlas
OS	Overall survival
UCEC	Uterine corpus endometrial carcinoma
CPTAC	Clinical Proteomic Tumor Analysis Consortium
UCSC	University of California Santa Cruz
FRGs	Ferroptosis-related genes
FerlncRNAs	Ferroptosis-related lncRNAs
FermiRNAs	Ferroptosis-related miRNAs
DEFRGs	Differentially expressed FRGs
DEFerlncRNAs	Differentially expressed FerlncRNAs
DEFermiRNAs	Differentially expressed FermiRNAs
log FC	Log fold change
PPI	Protein–protein interactions
K–M	Kaplan–Meier
ROC	Receiver operating characteristic
PCA	Principal component analysis
t-SNE	t-distributed stochastic neighbor embedding
DCA	Decision curve analysis
GSEA	Gene set enrichment analysis
CDKN2A	Cyclin-dependent kinase inhibitor 2A
AURKA	Aurora kinase A
LCN2	Lipocalin 2
AQP5	Aquaporin 5
SLC40A1	Solute carrier family 40 member 1
FANCD2	Fanconi anemia complementation group D2
DPP4	Dipeptidyl peptidase 4
AUC	Area under the curve
